# Decellularized extracellular matrix derived from dental pulp stem cells promotes gingival fibroblast adhesion and migration

**DOI:** 10.1186/s12903-024-04882-7

**Published:** 2024-10-01

**Authors:** Nunthawan Nowwarote, Zakaria Chahlaoui, Stephane Petit, Lucas T. Duong, Florent Dingli, Damarys Loew, Ajjima Chansaenroj, Chatvadee Kornsuthisopon, Thanaphum Osathanon, Francois Come Ferre, Benjamin P.J. Fournier

**Affiliations:** 1 Centre de Recherche des Cordeliers, Molecular Oral Pathophysiology, INSERM UMRS 1138, Université Paris Cité, Sorbonne Université, Paris, 75006 France; 2 Department of Oral Biology, Dental Faculty Garancière, Université Paris Cité, Paris, 75006 France; 3 Centre de Recherche, CurieCoreTech Spectrométrie de Masse Protéomique, Institut Curie, PSL Research University, Paris, France; 4 Center of Excellence for Dental Stem Cell Biology, Faculty of Dentistry, Chulalongkorn University, Bangkok, 10330 Thailand; 5 Department of Anatomy, Faculty of Dentistry, Chulalongkorn University, Bangkok, 10330 Thailand

**Keywords:** Decellularized extracellular matrix, Dental pulp stem cells, Gingival fibroblasts, Cell adhesion, Cell migration

## Abstract

**Background:**

Decellularized extracellular matrix (dECM) has been proposed as a useful source of biomimetic materials for regenerative medicine due to its biological properties that regulate cell behaviors. The present study aimed to investigate the influence of decellularized ECM derived from dental pulp stem cells (DPSCs) on gingival fibroblast (GF) cell behaviors. Cells were isolated from dental pulp and gingival tissues. ECM was derived from culturing dental pulp stem cells in growth medium supplemented with ascorbic acid. A bioinformatic database of the extracellular matrix was constructed using Metascape. GFs were reseeded onto dECM, and their adhesion, spreading, and organization were subsequently observed. The migration ability of the cells was determined using a scratch assay. Protein expression was evaluated using immunofluorescence staining.

**Results:**

Type 1 collagen and fibronectin were detected on the ECM and dECM derived from DPSCs. Negative phalloidin and nuclei were noted in the dECM. The proteomic database revealed enrichment of several proteins involved in ECM organization, ECM–receptor interaction, and focal adhesion. Compared with those on the controls, the GFs on the dECM exhibited more organized stress fibers. Furthermore, cultured GFs on dECM exhibited significantly enhanced migration and proliferation abilities. Interestingly, GFs seeded on dECM showed upregulation of *FN1*, *ITGB3*, and *CTNNB1* mRNA levels.

**Conclusions:**

ECM derived from DSPCs generates a crucial microenvironment for regulating GF adhesion, migration and proliferation. Therefore, decellularized ECM from DPSCs could serve as a matrix for oral tissue repair.

**Supplementary Information:**

The online version contains supplementary material available at 10.1186/s12903-024-04882-7.

## Introduction

The human oral cavity is a dynamic and complex environment with numerous cell types that contribute to its structure and function. The maintenance of oral tissue integrity and repair following injury or disease relies heavily on the intricate interplay between various cell populations and the extracellular matrix (ECM) [1]. The ECM, a complex network of proteins and glycoproteins, plays a pivotal role in regulating cellular behaviors under both physiological and pathological conditions, such as adhesion, migration, proliferation, and differentiation [2]. Recent advancements in regenerative medicine have led to the exploration of decellularized extracellular matrix (dECM) as a potent biomaterial for tissue engineering applications [3]. Derived from various tissue sources, dECM offers a complex and biologically active scaffold that closely replicates the native tissue microenvironment, thereby facilitating cellular responses [4].

Dental pulp stem cells (DPSCs) have garnered significant attention in recent years due to their remarkable regenerative potential and their ability to synthesize a unique ECM niche within dental pulp tissues [5]. The dECM derived from dental pulp tissue holds great promise for enhancing oral tissue regeneration, as it is anticipated to provide a natural microenvironment that closely mimics the native tissue ECM [6, 7]. Recent studies have illustrated the regenerative potential of decellularized extracellular matrix (dECM) obtained from DPSCs for bone tissue regeneration, as it promotes mineralization [8]. In addition, dECM generated from immobilized Jagged1-treated hDPSCs increases the odonto/osteogenic differentiation of stem cells isolated from the apical papilla (SCAPs) [9].

Understanding how this bioengineered matrix influences cellular responses, particularly cell adhesion and migration, is crucial. This knowledge is essential for advancing our understanding of oral tissue regeneration. Gingival fibroblasts, a primary cell type within periodontal tissues, play a pivotal role in maintaining gingival integrity and orchestrating the wound-healing process in response to injury or disease [10, 11]. This study aimed to investigate the effects of dECM derived from DPSCs on gingival fibroblast adhesion and migration. By elucidating the interactions between this biomaterial and resident oral cells, we aspire to uncover valuable insights into the potential applications of DPSC-derived dECM in promoting tissue repair, wound healing, and regenerative therapies within the oral cavity.

## Materials and methods

### Tissue explants and cell culture

Teeth were obtained from the dental surgery department at Charls Foix Hospital (Ethical no. CER-2021-038, ORCELL) using an aseptic procedure. Healthy patients aged 20–25 years were selected following ethical approval. Gingival and dental pulp tissues were chopped into small pieces and placed on tissue culture dishes. The explanted cells were cultured in an incubator at 37 °C and 5% CO_2_. The cells were maintained in DMEM (Eagle’s minimal essential medium) supplemented with 10% fetal bovine serum, 100 U/ml penicillin, 100 µg/ml streptomycin, 1% essential amino acid, and 0.25 µg/ml amphotericin B. After confluence, trypsin EDTA was used to detach the cells, and the cells were subcultured at a ratio of 1:3. The medium was changed every two days. Primary cells at passages 3–6 were used in the experiments.

### Extracellular matrix decellularization

The cells were cultured in growth medium for 21 days, and on day 7, the culture conditions were supplemented with 50 µg/ml L-ascorbic acid. After 21 days, decellularization was performed using 0.5% Triton X-100 in 20 mM ammonium hydroxide. To remove the remaining nucleotides, the samples were incubated for 30 min in 0.0025% DNase. After decellularization, the ECM was kept in PBS before being used in the experiments.

### Real-time polymerase chain reaction (real-time PCR)

Total RNA was extracted and purified using the RNeasy^®^ Mini Kit (Qiagen, Germany), and quantification was performed using the Quanti-iT™ RNA BR assay kit (Invitrogen, USA). One microgram of total RNA was converted to cDNA using Superscript™ II Reverse Transcriptase (Invitrogen, USA). Real-time PCR was performed using KAPA SYBR^®^ Fast Universal (Roche, Germany) with melt curve analysis for specificity on the CFX Connect Real-Time PCR system. The mRNA levels of the target genes were compared to those of the housekeeping gene (*SDHA*), and the comparative Ct method (2−∆Ct method) was used to measure relative gene expression. The primer sequences are shown in Table 1.

**Table 1:** Primer sequences

Genes	Accession Number	Sequence 5’-3’	Size (Base pair)
*CTNNB1*	NM_001904.4	Forward – GTG-CTA-TCT-GTC-TGC-TCT-AGT-A Reverse – CTT-CCT-GTT-TAG-TTG-CGA-CAT-C	154
*COLLAGEN 1A1*	NM_000088.4	Forward - AAC-CAA-GGC-TGC-AAC-CTG-GA Reverse - GGC-TGA-GTA-GGG-TAC-ACG-CAG-G	80
*FIBRONECTIN*	NM_212482.4	Forward - CGG-TGG-CTG-TCA-GTC-AAA-G Reverse - AAA-CCT-CGG-CTT-CCT-CCA-TAA	130
*INTREGIN ALPHA V*	NM_002205.5	Forward - GAC-AGG-GAA-GAG-CGG-GCA-CTA-TGG Reverse - GTC-CCT-TCC-CGG-CCG-GTA-AAA-CTC	231
*INTREGIN BETA I*	NM_002211.4	Forward - CCG-CGC-GGA-AAA-GAT-GAA-TTT Reverse - CCA-CAA-TTT-GGC-CCT-GCT-TG	130
*INTREGIN BETA III*	NM_000212.3	Forward - ACC-AGT-AAC-CTG-CGG-ATT-GG Reverse - CTC-ATT-GAA-GCG-GGT-CAC-CT	161
*KI67*	NM_002417.5	Forward – CGT-TTG-TTT-CCC-CAG-TGTC-T Reverse – CTC-CCT-GCC-CCT-TTC-TAT-TC	186
*SDHA*	NM_004168.4	Forward - AGC-AAG-CTC-TAT-GGA-GAC-CT Reverse - TAA-TCG-TAC-TCA-TCA-ATC-CG	200

### Immunofluorescence staining

The cells were incubated with 10% formaldehyde (Cat no. F8775, Sigma, USA) for 10 min. After washing with PBS, the samples were permeabilized with 0.1% Triton-X100 (Cat no. T9284, Sigma Aldrich, USA). The samples were blocked with nonspecific proteins in 10% BSA for 30 min and then incubated with primary antibodies against type I collagen (Cat no. ab34710, Abcam), fibronectin (HFN 7.1, DSHB), and rhodamine-phalloidin (Cat no. ab176753, Abcam). Phalloidin is a highly selective bicyclic peptide used for staining filamentous actin (F-actin). Goat anti-rabbit IgG Alexa Flour™ 594 or goat anti-mouse IgG Alexa Flour™ 594 was used as the secondary antibody. Cell nuclei were stained with DAPI (Cat no. R37606, Invitrogen, USA). A negative control was performed by eliminating the primary antibody incubation step. The staining was visualized under a fluorescence microscope (EVOS^®^ FL, Life Technology, USA).

### Extracellular matrix pellet digestion

ECM digestion was performed as described in a previous study [8]. Briefly, Millipore’s compartment protein extraction kit was used to extract the ECM. The ECM pellets were dissolved, followed by a series of digestion and enzymatic treatments involving urea, ammonium bicarbonate, DTT, iodoacetamide, PNGaseF, trypsin, and LysC at specific enzyme-to-substrate ratios.

### Bioinformatics analysis

Mass spectrometry (LC‒MS/MS) was performed as previously described [8]. The data were further processed using myProMS software [12]. The Human Matrisome Proteins dataset (Human Matrisome, Updated 2014) can be accessed at https://sites.google.com/uic.edu/matrisome/matrisome-annotations/homo-sapiens. This dataset contains proteins with at least two total peptides in all replicates. For Gene Ontology (GO) enrichment analysis, only proteins detected with five or more matching peptides were considered. The mass spectrometry proteomics data have been deposited in the ProteomeXchange Consortium (http://proteomecentral.proteomexchange.org) via the PRIDE partner repository [13] with the dataset identifier PXD018951.

### Cell migration assay

A scratch technique was performed. Briefly, cells were grown in 35-mm tissue culture dishes until confluence was achieved, after which a cell-free scratch 100–150 μm in width was created using a 200-mL pipette tip. A collagen type I (Cat no. 354236, BD Biosciences, USA) coating dish (at a concentration of 10 µg/ml) was used as the positive control. At 0, 12, 24, and 48 h, area closure was observed under a transmitted light microscope (EVOS^®^ FL, Life Technology, USA). The dishes were marked to identify viewpoints for the same area for capture at each time point. The free area was determined using ImageJ software as previously described [14]. The percentage of scratches that were close to each other was calculated.

### Cell proliferation assay

Gingival fibroblasts (12,500 cells) were seeded in 24-well plates (*n* = 4). The cells were treated with a 0.5 mg/mL MTT solution for 15 min at 37 °C. At the designated time points, the precipitated crystals were solubilized in dimethyl sulfoxide/glycine buffer. A microplate reader was used to measure the absorbance at 570 nm (BertholdTech TriStar, Driver Version: 1.07, (1.0.7.6), S/N: 25-1037, Embedded Version: 1.04, USA). The percentage of cells on the dECM was calculated and compared with the percentage of cells on the tissue culture plate.

### Colony formation assay

Gingival fibroblasts were seeded in six-well plates at 250 cells per well and maintained in growth medium for 14 days. The cells were fixed with 10% formaldehyde (Sigma, USA) for 10 min and stained with Coomassie^®^ Brilliant Blue G250 (Serva, UK). Colony formation was investigated using a transmitted light microscope (EVOS^®^ FL, Life Technology, USA).

### Statistical analysis

The data are presented as the means ± standard errors. The statistical analysis was performed using Prism 10 (GraphPad Software, CA, USA). Two-way analysis of variance (ANOVA) with the Geisser-Greenhouse correction was used to assess the differences between multiple groups, followed by Tukey’s multiple comparison test, followed by Tukey’s multiple comparison test, was used to assess the differences between multiple groups. A statistically significant difference was defined as *p* < 0.05, with higher values indicating significance. All statistical analyses were conducted with a sample size of *n* = 4 per group.

## Results

### Characterization of extracellular matrix and decellularized extracellular matrix derived from dental pulp stem cells

**Fig. 1:**
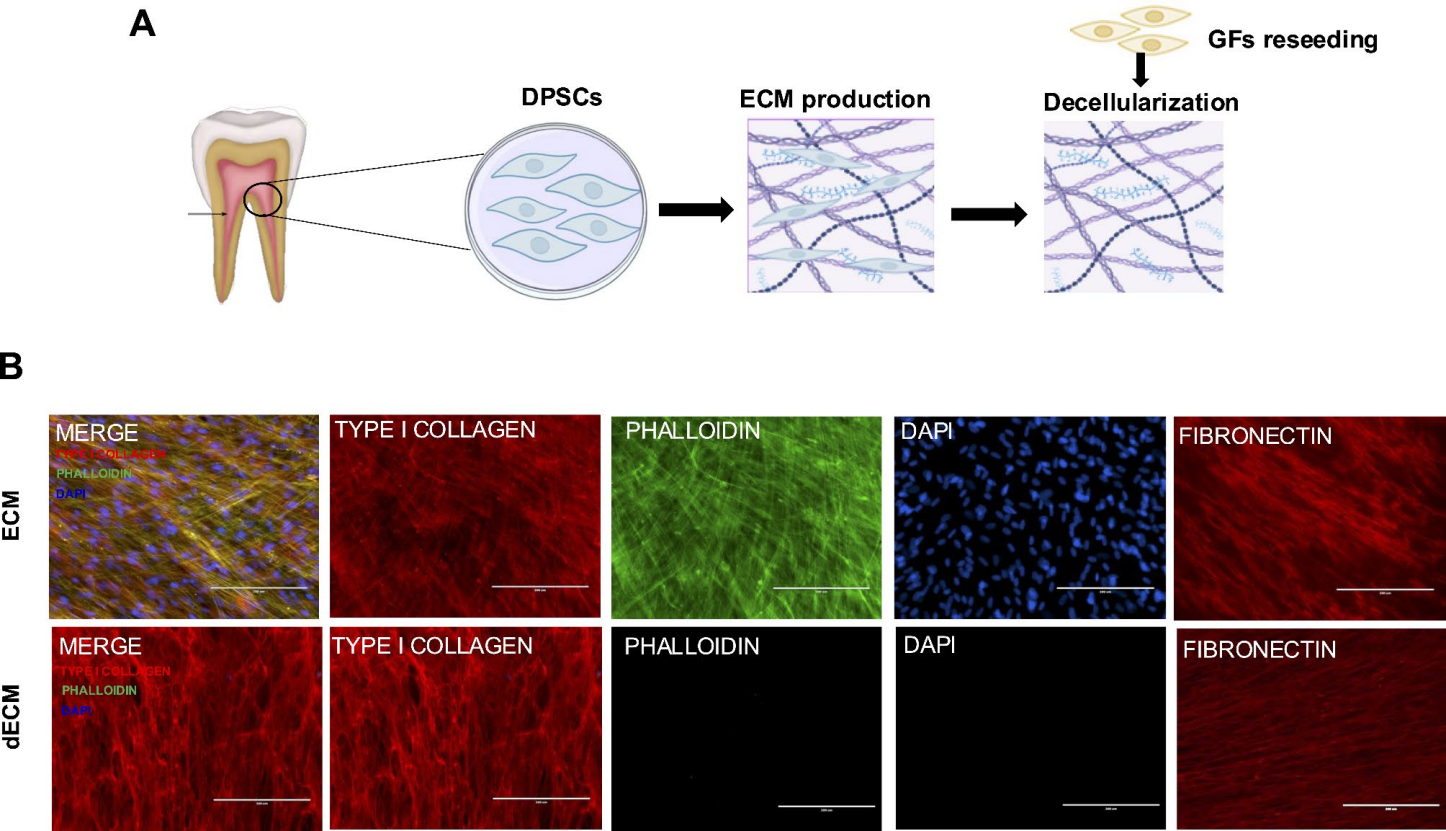
Characterization of the extracellular matrix and decellularized extracellular matrix. (**A**) Conceptual framework depicting the experiment from ECM production to reseeding of gingival fibroblasts (GFs) on decellularized ECM. (**B**) Both ECM samples exhibited positive staining for TYPE I COLLAGEN and FIBRONECTIN. The intracellular cytoskeleton and nuclei were visualized using phalloidin and DAPI, respectively. No residual cells were observed, as indicated by negative actin and DAPI staining in the dECM

Stem cells isolated from dental pulp tissues exhibit characteristics of mesenchymal stem cells (MSCs), including the ability to differentiate into osteogenic and adipogenic lineages. These cells expressed markers characteristic of mesenchymal stem cells but did not express surface markers typical of hematopoietic stem cells [8, 9, 15]. In the present study, we conducted a comprehensive characterization of the ECM and dECM, focusing on various key aspects to gain a deeper understanding of their composition and properties. The conceptual framework illustrates the process of extracellular matrix (ECM) synthesis and the subsequent reseeding of gingival fibroblasts on decellularized ECM (Fig. 1A). Analysis of the protein composition of both the ECM and dECM revealed significant differences. By day 21, both ECMs were found to contain a rich diversity of native ECM proteins, including type 1 collagen and fibronectin. In contrast, dECM exhibited a decreased presence of actin filament proteins due to the decellularization process. DNA content analysis confirmed the effective removal of cellular material in the dECM, resulting in significantly lower DNA levels than those in the native ECM (Fig. 1B). This indicates the successful elimination of potential immunogenic and cellular remnants.

### Enrichment protein analysis of the extracellular matrix from dental pulp stem cells

**Fig. 2:**
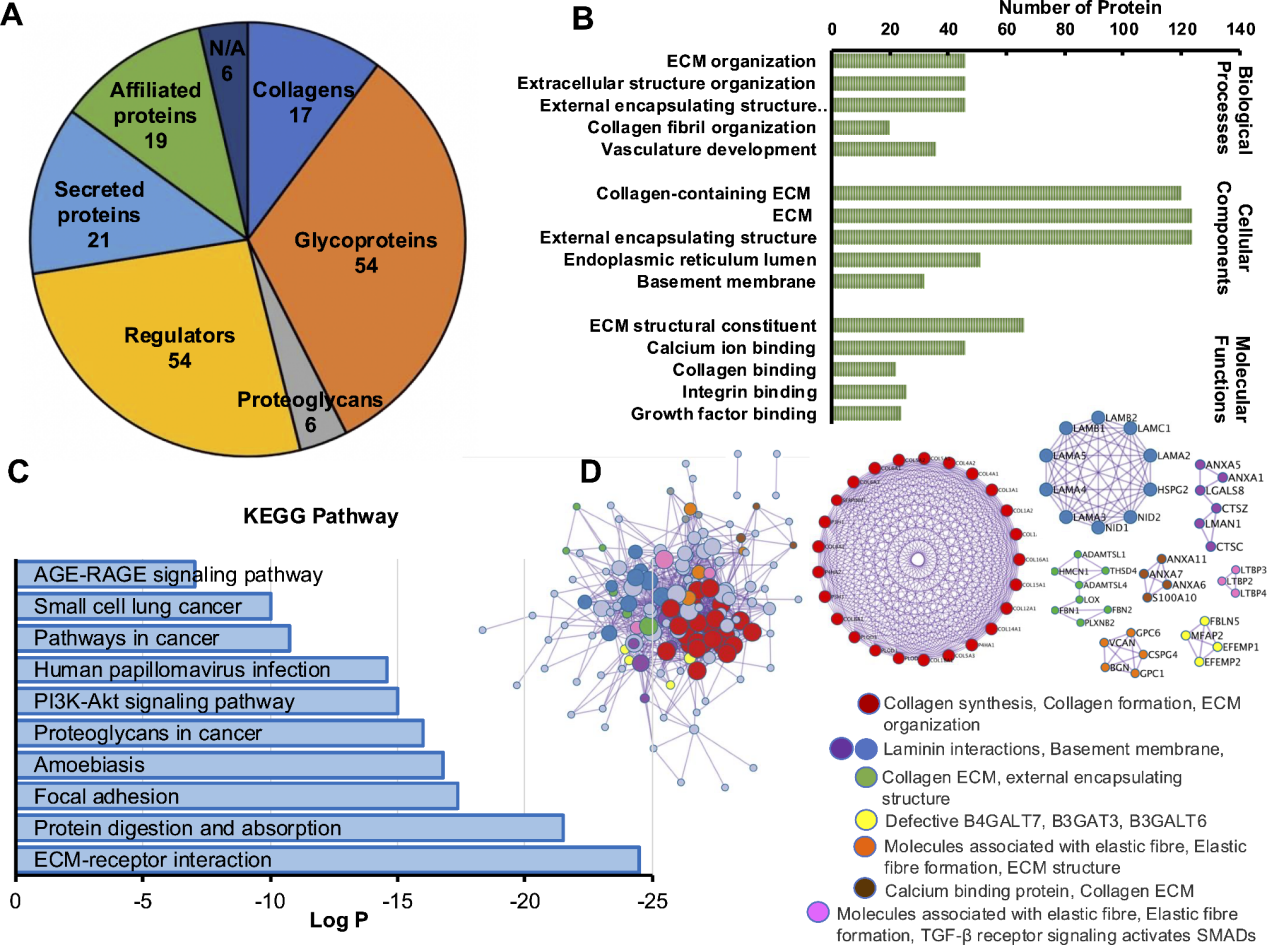
Enrichment protein analysis of extracellular matrix from dental pulp stem cells. (**A**) Matrix analysis of the ECM derived from human dental pulp stem cells (DPSCs). (**B**) Gene Ontology analysis of biological processes, cellular compartments, and cellular functions. (**C**) Log2 ratio of KEGG pathway enrichment analysis. (**D**) Protein‒protein interaction analysis identifying ECM proteins that interact

Protein enrichment analysis was performed to identify the specific proteins present in the ECM secreted by DPSCs. Cellular components were extracted from the ECM derived from DPSCs. The Matrisome Protein Database was used as the background for the protein list. A total of 167 different proteins were identified. The identified proteins included a total of 17 collagens, 54 glycoproteins, 6 proteoglycans, 44 proteins involved in regulating the extracellular matrix (ECM), 21 ECM-affiliated proteins, 19 secreted factors, and 6 proteins that have not yet been identified (Fig. 2A). The list of proteins contained in dECM derived from dental pulp stem cells are present in Table 2. The proteins identified in the matrisome database were subjected to pathway and gene ontology (GO) enrichment analysis. The results revealed enrichment of several proteins involved in collagen-containing extracellular matrix (cellular component), extracellular matrix structural constituent (molecular function), and extracellular matrix organization of cellular processes (biological process) (Fig. 2B). KEGG pathway enrichment analysis revealed that the proteins in the ECM derived from DPSCs were associated with ECM-receptor interactions, protein digestion and absorption, and focal adhesion (Fig. 2C). Subsequently, protein‒protein interactions (PPIs) were analyzed to explore how the identified ECM proteins interact with each other and with cellular proteins. This analysis aimed to provide insights into the intricate molecular networks involved in ECM-mediated cellular responses. All protein‒protein interactions among the input proteins were extracted from the PPI data source to construct a PPI network. A total of 141 ECM proteins derived from DPSCs interacted with 14 MCODEs. Furthermore, the MCODE algorithm was then applied to this network to identify neighborhoods where proteins were densely connected (Fig. 2D).

**Table 2:** Matrisome protein list of extracellular matrix protein derived from dental pulp stem cells

Core Matrisome				Matrisome assosiated protein				
Collagens	ECM Glycoproteins		Proteoglycans	ECM Regulators		Affiliated Proteins	Secreted Factors	*N*/A
COL12A1	AEBP1	LAMB1	HSPG2	A2M	LOXL1	ANXA1	ANGPTL2	AIFM2
COL14A1	CRELD1	LAMB2	VCAN	ADAM10	LOXL2	ANXA11	CCBE1	C19orf10
COL15A1	CTGF	LAMC1	HAPLN1	ADAM17	MASP1	ANXA13	CXCL12	FGFR1
COL16A1	CTHRC1	LTBP1	DCN	ADAM9	MMP14	ANXA2	FSTL1	P3H1
COL18A1	CYR61	LTBP2	BGN	ADAMTSL1	MMP2	ANXA4	GDF15	P3H3
COL1A1	ECM1	LTBP3	LUM	ADAMTSL4	P4HA1	ANXA5	HCFC1	STK4
COL1A2	EDIL3	LTBP4		CD109	P4HA2	ANXA6	HGF	
COL3A1	EFEMP1	MFAP2		CSTB	PLAT	ANXA7	IGF2	
COL4A1	EFEMP2	MFGE8		CTSA	PLAU	COLEC12	MDK	
COL4A2	EMILIN1	MXRA5		CTSB	PLOD1	CSPG4	PTN	
COL5A1	FB + A20:A73N1	NID1		CTSC	PLOD2	GPC1	S100A10	
COL5A2	FBLN1	NID2		CTSD	PLOD3	GPC6	S100A11	
COL5A3	FBLN2	PCOLCE		CTSK	PRSS1	GREM1	S100A13	
COL6A1	FBLN5	POSTN		CTSL	SERPINB1	LGALS1	S100A16	
COL6A2	FBN2	PXDN		CTSZ	SERPINB6	LGALS3	S100A6	
COL6A3	FBN3	SLIT2		F13A1	SERPINE2	LGALS8	SFRP1	
COL8A1	FN1	SLIT3		F2	SERPINF1	LMAN1	TGFB2	
	GAS6	SNED1		HTRA1	SERPINH1	PLXDC2	WNT5A	
	HMCN1	SPARC		HTRA3	TGM2	PLXNB2	WNT5B	
	IGFBP2	SRPX		ITIH2	TIMP1	PLXND1		
	IGFBP4	SVEP1		ITIH3	TIMP2	SEMA3C		
	IGFBP5	TGFBI		LOX	TIMP3			
	IGFBP7	THBS1						
	LAMA2	THBS2						
	LAMA3	THSD4						
	LAMA4	TNC						
	LAMA5	VWA5A						

### Increased attachment and spreading of gingival fibroblasts by decellularized extracellular matrix derived from dental pulp stem cells

**Fig. 3:**
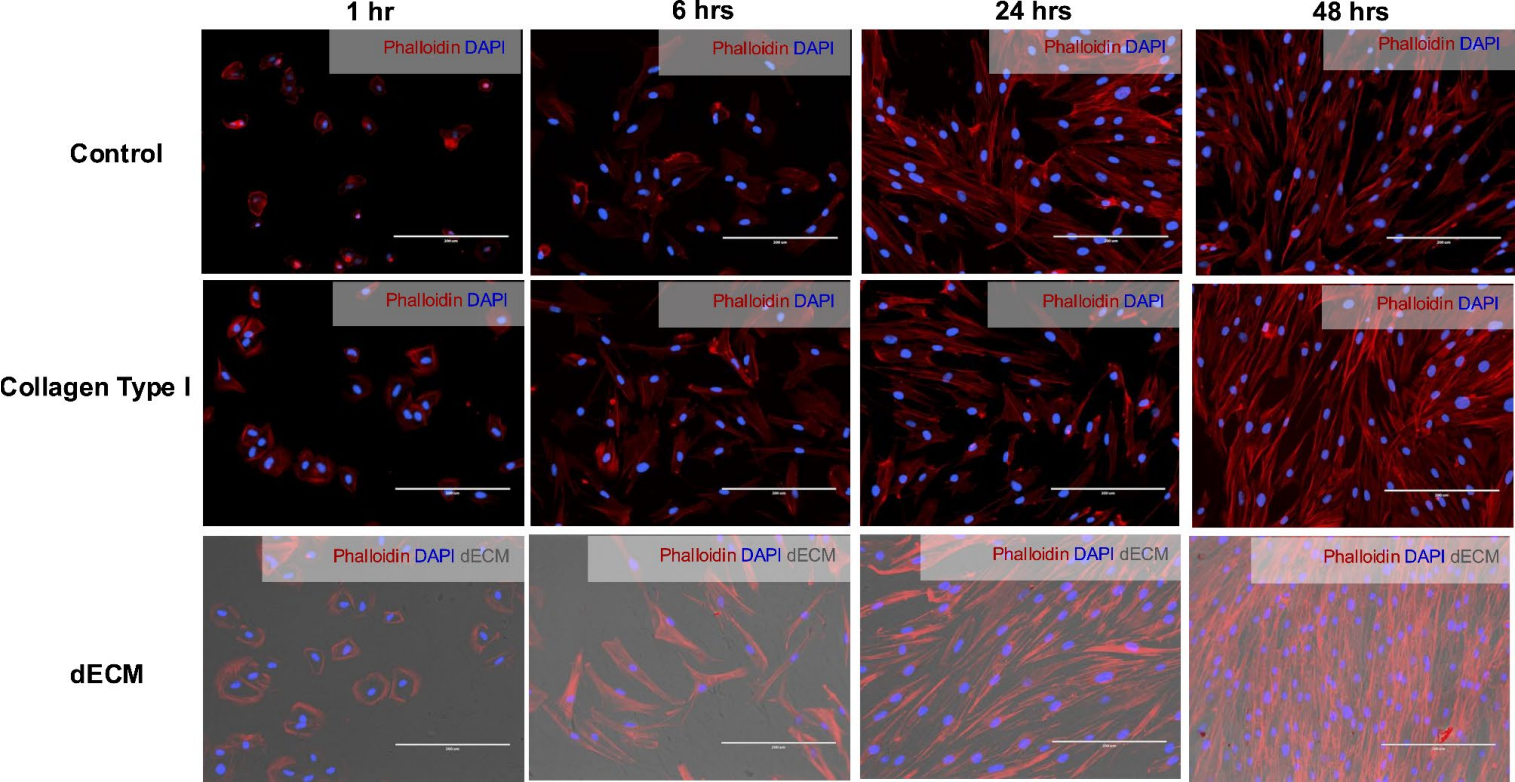
Influence of decellularized extracellular matrix derived from dental pulp stem cells on gingival fibroblast adhesion. Microscopy images of GFs cultured on glass (control), collagen type I-coated, and dECM-coated substrates. F-actin was stained with rhodamine-phalloidin. Scale bars represent 200 μm

In our study, we investigated the influence of dECM derived from DPSCs on the behavior of oral cells, focusing on cell attachment and spreading. Gingival fibroblasts were seeded on glass as a negative control, while a glass coating of collagen type I was utilized as a positive control. Our findings indicate a significant enhancement in both oral cell attachment and spreading when exposed to dECM compared to control conditions (Fig. 3). After seeding for one hour, gingival fibroblasts were able to attach to the culture substrate. Furthermore, gingival fibroblasts exposed to DPSC-derived dECM exhibited a notable increase in cell spreading. Cells in contact with the dECM displayed a more flattened and spread-out morphology than cells in the control group, which typically appeared more rounded and less adherent. Moreover, after 48 h, strong and intense staining of actin filaments was observed in the cells on the dECM compared to the control. This vivid staining indicated that a significant proportion of the cultured cells were firmly attached to the substrate.

### Enhancement of gingival fibroblast migration by decellularized extracellular matrix derived from dental pulp stem cells

**Fig. 4:**
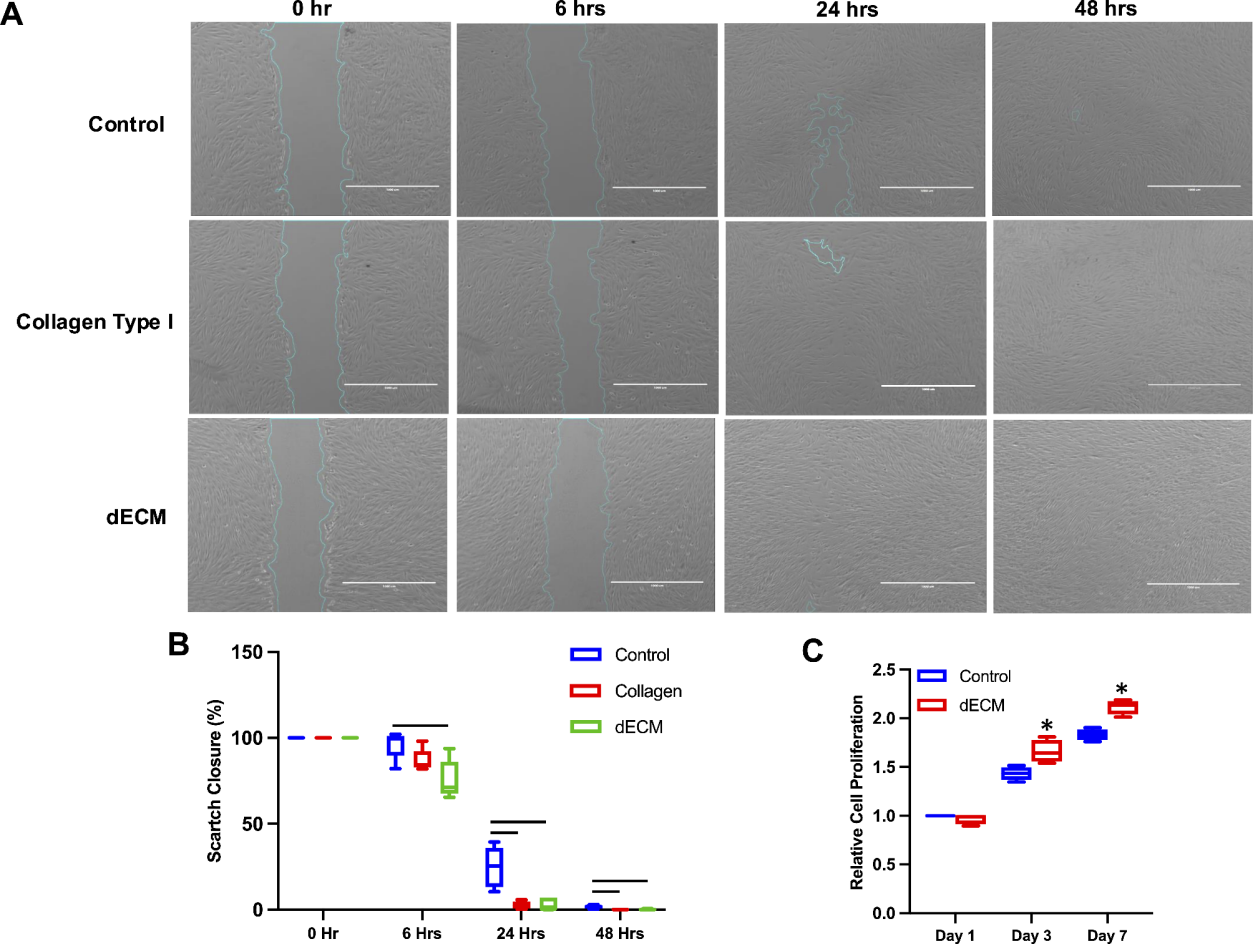
The impact of decellularized extracellular matrix derived from dental pulp stem cells on gingival fibroblast migration. The in vitro scratch experiment depicted GFs cultured on glass (control), collagen type I-coated (positive control), and dECM-coated substrates for 0, 6, 24, and 48 h. Scale bars represent 1000 μm. (**B**) Quantitative analysis revealed a significant decrease in the scratch closure of gingival fibroblasts cultured on dECM compared to control conditions at 0, 6, 24, and 48 h. (**C**) The graph depicts the proliferation rate of GFs cultured on dECM at days 1, 3, and 7. Statistical significance is denoted by asterisks, and bars indicate a significant difference between groups (*p* < 0.05)

In our study, we investigated the impact of dECM derived from DPSCs on oral cell behavior, particularly focusing on the migratory and proliferative capabilities of these cells. Tissue culture dishes coated with collagen type I served as the positive control. Our findings revealed a significant enhancement in gingival fibroblast migration in the DPSC-derived dECM group compared to the control group (Fig. 4A). Quantification was performed via a scratch closure assay (Fig. 4B). Compared with that of the control group, the scratch closure of gingival fibroblasts seeded on dECM was significantly different at 6 h. At 24 and 48 h, a significant difference in the wound closure area was notably observed between the dECM and collagen type I conditions compared to the control condition.

### Enhancement of gingival fibroblast proliferation by decellularized extracellular matrix derived from dental pulp stem cells

**Fig. 5:**
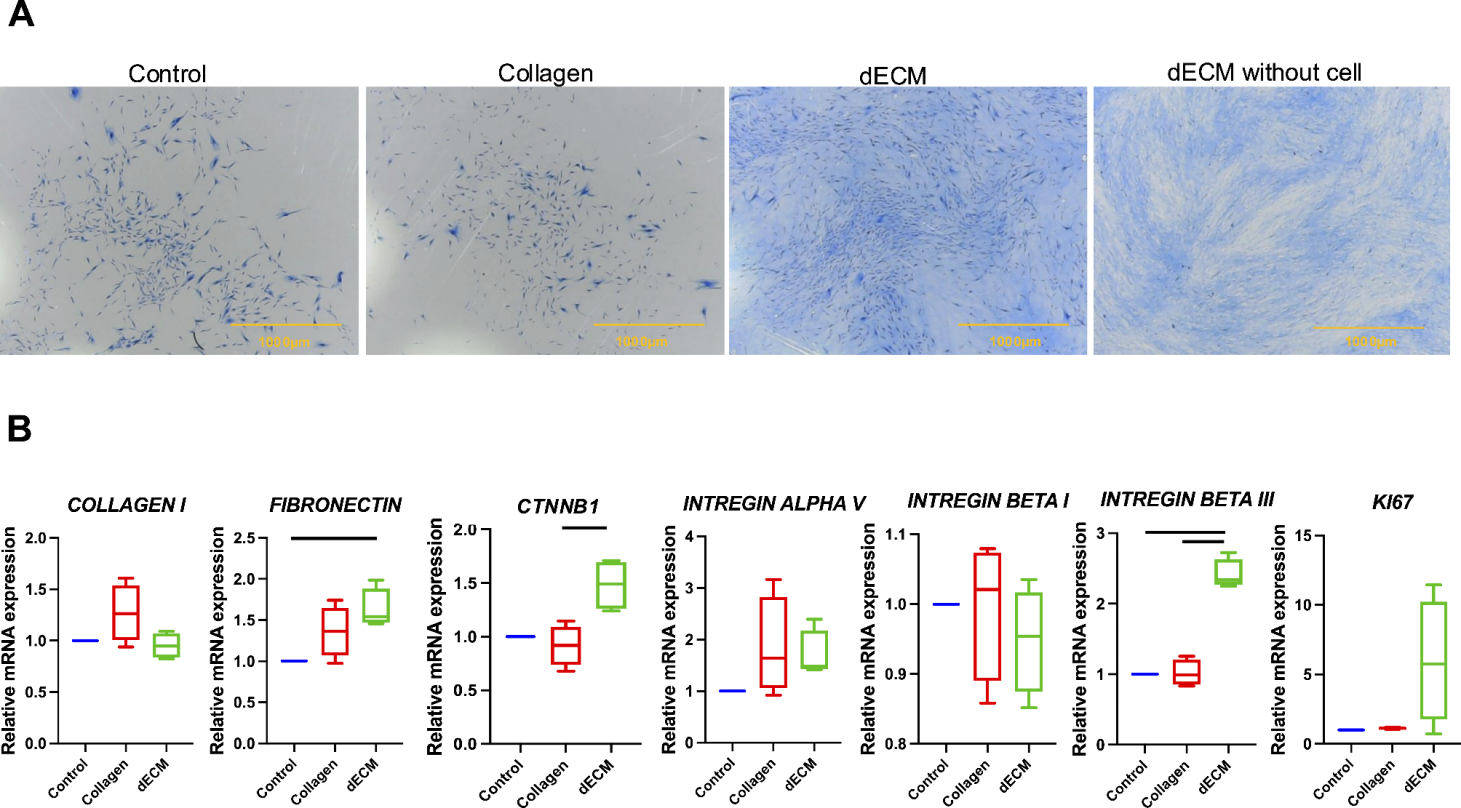
The effects of decellularized extracellular matrix derived from dental pulp stem cells (DPSCs) on gingival fibroblast proliferation. (**A**) Colony-forming unit cell morphology is represented by the colorization of Coomassie blue, while dECM without cell staining was used to distinguish between protein staining and the actual cell-forming colonies. (**B**) mRNA expression of GFs seeded on dECM at 48 h. Scale bars represent 1000 μm. The data are presented as the means ± standard errors (SEs). Statistical significance is denoted by bars, indicating a significant difference between groups (*p* < 0.05)

In our study, we assessed whether the presence of DPSC-derived dECM influenced the ability of oral cells to form colonies, a critical indicator of cell self-renewal and proliferation potential. Our findings revealed that gingival fibroblasts cultured in the presence of dECM exhibited a significant increase in cell viability at days 3 and 7 compared to those cultured in the control condition (Fig. 4C), indicating sustained cellular health and suggesting proliferation. Surprisingly, our analysis did not reveal any significant difference in the colony-forming unit ability of gingival fibroblasts exposed to DPSC-derived dECM compared to those in the control group. All groups displayed a similar capacity to form colonies. To distinguish between protein staining and the actual cell-forming colonies, dECM without cell staining was used (Fig. 5A).

### Upregulation of mRNA expression in gingival fibroblasts induced by decellularized extracellular matrix derived from dental pulp stem cells

We investigated the impact of DPSC-derived dECM on the mRNA expression levels of specific genes in gingival fibroblasts. This analysis aimed to understand the molecular signaling pathways and cellular processes influenced by the interaction of gingival fibroblasts with the dECM. The results showed that after 48 h, gingival fibroblasts seeded on dECM significantly upregulated the mRNA expression of *FN1*, *ITGB3*, and *CNNTB1* compared to that in the control group. Additionally, the gene marker of cell proliferation, *KI67*, showed a trend toward increased mRNA expression (Fig. 5B).

## Discussion

This work aimed to investigate the potential of dental pulp stem cell-derived decellularized extracellular matrix (dECM) for promoting the adhesion and migration of gingival fibroblasts. Our study aimed to comprehensively understand the interactions between the dECM and gingival fibroblasts. The presence of collagen type I and fibronectin in the ECM and dECM derived from DPSCs indicates the composition and characteristics of these matrices. Additionally, the observation of negative intracellular proteins and nuclei within the dECM highlights the successful removal of cellular components during the decellularization process. Proteomic database analysis revealed enrichment of several proteins involved in ECM organization, ECM-receptor interaction, and focal adhesion. These findings shed light on the molecular composition and functional characteristics of the ECM derived from DPSCs.

Understanding how this bioengineered matrix affects cell adhesion and migration is essential for oral tissue regeneration research. GFs were evaluated in comparison to the controls. In our study, we found that collagen type I is abundant in both the ECM and dECM derived from DPSCs. Using collagen type I-coated dishes as a positive control enabled us to evaluate the performance of the dECM. The stress fibers of gingival fibroblasts grown on dECM had a greater degree of organization. In addition, gingival fibroblasts demonstrated a greatly improved capacity for migration and proliferation when grown on dECM. It is likely that gingival fibroblasts grown on dECM exhibit increased mRNA expression of genes involved in the biological functional impact of dECM.

Our previous studies demonstrated that ECM derived from DPSCs expressed ECM proteins in both the ECM and dECM. Decellularization involves removing cellular components while preserving the structural integrity and bioactive molecules of the ECM [8, 9]. The successful removal of these intracellular proteins ensures that the dECM is free from cellular remnants, reducing potential immunological reactions and making it suitable for various tissue engineering applications [16]. Decellularization using chemicals including Triton X-100 and sodium dodecyl sulfate (SDS), provide effective elimination of cellular component. Compared to Triton X100, SDS has several disadvantages. Residual amounts of SDS left in the dECM can cause an increased inflammatory response and decreased cell viability [17]. Additionally, SDS was found to damage the ECM microstructure, destroy the ECM laminar array, remove most of the sulfated glycosaminoglycans (sGAG), and affect the integrity of growth factors and cytokines [18]. Triton X-100 is generally less damaging to the extracellular matrix structure and retains bioactive molecules better than SDS [19]. In addition, Triton X-100 has been reported to be the most effective decellularization agent for ligament tissue engineering [20] and human kidney bioengineering using human mesenchymal stem cells [21]. Therefore, in the present study, we employed Triton X-100 for decellularization. Type I collagen, a fundamental component of the ECM, provides structural support and helps maintain tissue integrity [1]. Its presence in both the ECM and dECM indicates that DPSCs can produce this crucial protein, which is essential for tissue structure and function. Fibronectin contributes to the ability of the matrix to support cell behavior, such as adhesion and spreading.

In addition, limitations such as ECM protein loss, incomplete decellularization due to high confluence, variations in chemical concentration and incubation time, ECM disruption before recellularization, and potential cytotoxicity or immunological responses have been identified [22]. Enrichment analysis of ECM proteins was conducted using samples from three different donors. A total of 163, 116, and 51 proteins were detected in the respective samples, 47 of which were common (Supplemental Fig. 1A). The loss of ECM proteins during the decellularization process did not significantly affect the enrichment of protein‒protein interactions (Supplemental Fig. 1B). Furthermore, the expression of ECM proteins in DPSCs is influenced by the conditions of the donor teeth. Compared with immature dental pulp cells, mature dental pulp cells exhibit stronger expression of collagen type I, fibronectin, and tenascin, while vimentin shows consistent expression levels [23]. Hence, variations in ECM protein expression among patients may be attributed to genetic differences [24]. The concept of patient-specific ECM signatures underscores the uniqueness of each individual’s ECM composition and warrants further investigation.

In this study, we utilized the matrisome protein database as a reference [19] to identify 167 different proteins, aiming to determine the composition of the ECM derived from DPSCs. Glycoproteins were the most abundant, followed by regulator proteins, affiliated proteins, secreted proteins, collagens, and proteoglycans. Similarly, our previous study identified 225 matrisome proteins with comparable patterns in the matrisome category [8]. In the periodontal ligament ECM, 119 matrisome proteins were found, with regulator proteins, glycoproteins, collagens, associated proteins, proteoglycans, and secretory proteins being the most prevalent [25]. Conversely, the oral epithelium, as well as the early and late stages of oral tumors, exhibited 76, 63, and 43 matrisome proteins, respectively, with collagens, glycoproteins, proteoglycans, regulator proteins, affiliated proteins, and secreted proteins showing the greatest enrichment [26]. These findings collectively suggest that the distinct ECM composition of each cell type or tissue directly influences its function.

The enrichment protein analysis of the ECM derived from DPSCs represents a crucial step in understanding the composition and functional significance of this specialized microenvironment. This analysis has provided valuable insights into the repertoire of ECM proteins, including structural proteins such as collagen, glycoproteins, and proteoglycans [27]. The significant enrichment of proteins related to cellular components in the gene ontology analysis indicated that ECM-derived proteins are involved in building and maintaining various structural elements within cells [28]. The number of proteins identified through gene ontology enrichment was noted in cellular components, cellular functions, and biological processes. Similarly, protein‒protein interactions were observed in collagen synthesis, collagen formation, ECM organization, laminin interactions, and the basement membrane. The recognition of laminin by integrin receptors is one of the most essential receptor types that mediates cell attachment to ECM components [29]. Our KEGG pathway enrichment analysis revealed several pathways related to cell adhesion and migration, including ECM-receptor interactions [30] and PI3K-Akt signaling pathway [31]. ECM directly communicates with cells through cell surface receptors, mainly integrins, which initiate downstream intracellular signaling and control various cellular functions [32]. Therefore, an increase in ECM-receptor interactions would be beneficial in promoting cell proliferation, adhesion, and migration of reseeded GFs. Taken together, these results suggest that dECM functions are essential for cellular responses to the ECM environment and influence cell behavior. However, further investigation using inhibitors against signaling pathways, such as integrins (a family of cell surface receptors involved in cell adhesion that link the ECM to the cell cytoskeleton via focal adhesions) and the PI3K-Akt pathway, should be conducted to study the in-depth intracellular mechanisms of dECM-mediated cell adhesion and migration.

The ECM controls cell behaviors during development, growth, and tissue regeneration [33]. ECM component compositions regulate tissue stiffness, elasticity, and permeability [34]. The current study demonstrated that the utilization of dECM substantially enhanced the adhesion, migration, and proliferation of gingival fibroblasts. Similarly, dECM derived from various sources of MSCs, such as adipose tissue [35], bone marrow (BM) [36–38], cartilage [39] and the umbilical cord [40], has been found to improve cell adhesion, migration, and proliferation. In addition, dECM derived from human periodontal ligament stem cells (hPDLSCs) promoted DPSC adhesion and spreading, as indicated by the strong expression of a focal adhesion protein (vinculin) and cell proliferation [41]. However, the lack of significant effects on colony-forming ability raises interesting questions and considerations. A previous study reported that the colonies of adipose stem cells were larger and had greater cell density when cultured on ECM derived from BM. Compared with cells at late passages (passage 5), cells at lower passages (passage 3) exhibited greater colony-forming ability [37]. Compared with cells cultured on medium without serum, cells cultured on BM-ECM in the presence of serum at passage 1 had significantly greater colony-forming units and proliferation rates [42]. These findings suggest that a unique microenvironment promotes the colony-forming capacity and proliferation of MSCs while preserving their stem cell characteristics. Therefore, DPSC-derived dECM may enhance individual cell adhesion, migration, and proliferation; however, it may not substantially impact the ability of gingival fibroblasts to form colonies from single cells. The enhancement of gingival fibroblast attachment by dECM derived from DPSCs is a significant finding with implications for oral tissue regeneration. Gingival fibroblasts play a crucial role in maintaining gingival integrity and wound healing in the oral cavity [43].

The presence of specific ECM proteins, such as fibronectin and collagen, provides adhesive cues for cells, facilitating their attachment to the ECM and surrounding tissues. The identification of signaling focal contacts mediated by integrin beta3 (ITGβ3) has revealed its role in facilitating cell adhesion and migration, as well as its ability to mediate ECM-derived physical cues from bone marrow to modify the activity of hematopoietic stem and progenitor cells (HSPCs) [44]. Together, laminin-binding integrins encourage epithelial cell attachment and proliferation [45]. Moreover, the specific binding of ITGβ3 to the fibronectin ligand promoted the long-term repopulation of stem cells [46]. Correspondingly, greater PDL cell proliferation and migration were triggered by the presence of fibronectin [47]. Consistently, our results showed that ITGB3 and FN1 mRNA expression was significantly upregulated in gingival fibroblasts cultured on dECM derived from DPSCs.

WNT signaling pathways promote pulp healing, dentin repair, and stemness-related epigenetic regulation [48]. Wnt/β-catenin signaling is activated during dental pulp healing and repair [49]. Activation of this pathway can promote tissue regeneration, making it a crucial target for studies related to wound healing and regeneration [50]. Our results demonstrated that *CTNNB1* (the gene name for β-catenin) mRNA expression increased in gingival fibroblasts cultured on dECM derived from DPSCs. Upregulation of this gene in our study is correlated with previous report stating that MSCs seeded on dECM derived from urine-derived stem cells enhanced chondrogenic differentiation via the WNT signaling pathway [51]. Given the fact that β-catenin is the hallmark of canonical WNT signaling, enhanced expression of this gene in dECM group suggested that WNT signaling might be one of the critical pathways through which the ECM regulates cellular responses. Integrins are essential cell surface receptors involved in ECM-mediated cellular responses [52]. Their enhanced expression in the dECM group correlates with pathway enrichment analysis, indicating that dECM promotes ECM-receptor interactions. Taken together, these findings suggest that interactions between the laminin basement dECM and β3 integrin could play a role in the adhesion, migration, and proliferation of gingival fibroblasts on the dECM, possibly involving the WNT signaling pathway. This finding highlights the complex interplay between the ECM microenvironment and cellular signaling and its relevance for tissue regeneration and wound healing in the oral cavity. Further research is necessary to elucidate the specific molecular pathways and mechanisms involved in gingival fibroblast adhesion, migration, and proliferation induced by DPSC-derived dECM and to explore their clinical applications in the context of oral health and regenerative medicine.

## Conclusion

This study investigated how DPSC-derived dECM enhances gingival fibroblast adhesion, migration, and proliferation. Our findings illuminate the pathway through which DPSC-derived dECM influences gingival fibroblast responses, revealing its capacity to support crucial cellular processes involved in oral tissue repair.

## Electronic supplementary material

Below is the link to the electronic supplementary material.

Supplementary Material 1

## Data Availability

The mass spectrometry proteomics data have been deposited in the ProteomeXchange Consortium (http://proteomecentral.proteomexchange.org) via the PRIDE partner repository with the dataset identifier PXD018951.
