# Supplementary figures and images for: Decellularized extracellular matrix derived from dental pulp stem cells promotes gingival fibroblast adhesion and migration
Source: BMC Oral Health. 2024 Oct 1;24:1166. doi: 10.1186/s12903-024-04882-7 (PMC11443845; doi:10.1186/s12903-024-04882-7)

Supplement Figure 1.

A

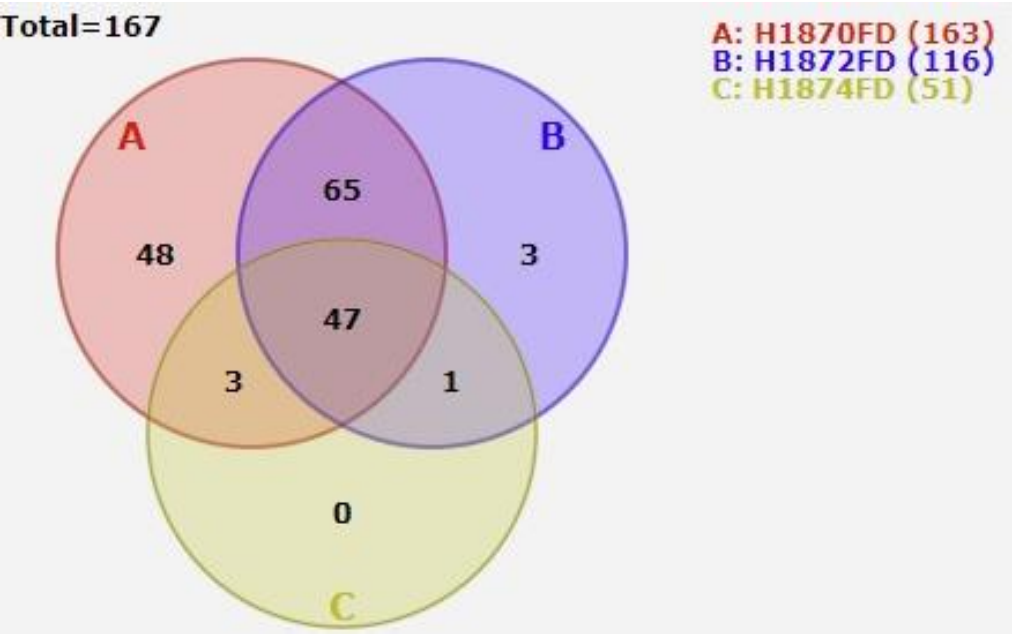

B 47 Common proteins and 3 MCODES

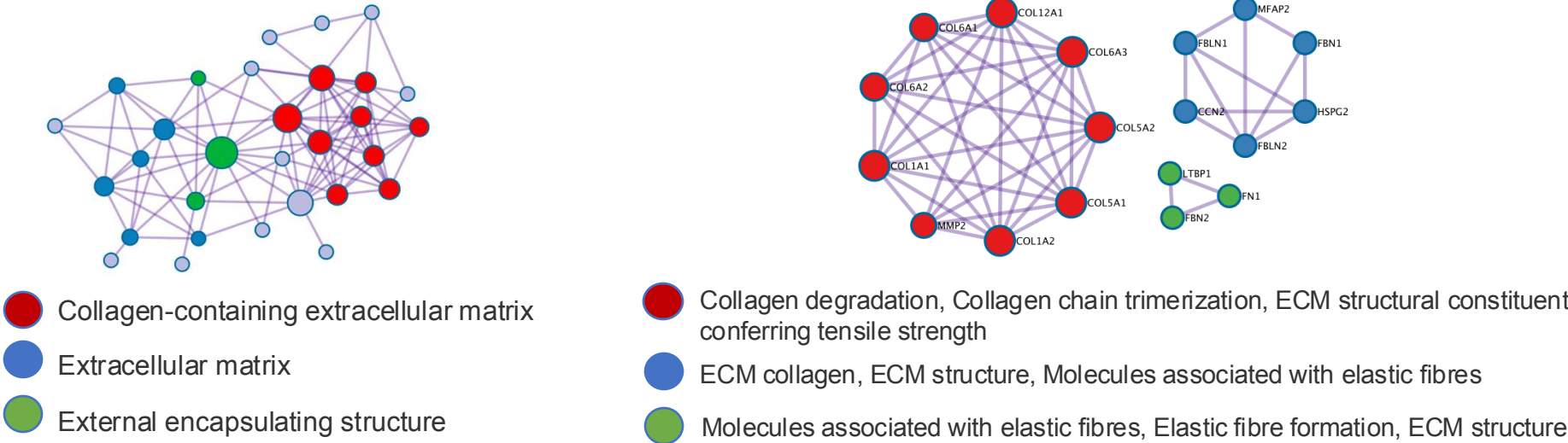

Supplement: Supplementary file 1 — Supplementary Material 1 [file 12903_2024_4882_MOESM1_ESM.pdf]
